# Gene expression asymmetry in Parkinson’s disease: variation of *CCT* gene expression is correlated with hemisphere specific severity

**DOI:** 10.3389/fnmol.2025.1743557

**Published:** 2026-01-13

**Authors:** Steven E. Pierce, Edwin J. C. van der Schans, Thomas M. Goralski, Elizabeth Ensink, Peipei Li, Michael X. Henderson, Gerhard A. Coetzee

**Affiliations:** Department of Neurodegenerative Science, Van Andel Institute, Grand Rapids, MI, United States

**Keywords:** asymmetry, brain lateralization, CCT, chaperonin, Parkinson’s disease

## Abstract

Parkinson’s disease (PD) symptom onset is typically unilateral, which may be related to molecular differences underlying hemispheric vulnerability. Here we sampled prefrontal cortex bilaterally from people with PD and healthy controls and performed RNA-seq on neuronal nuclei to determine hemispheric and disease-related differences. Brain hemispheres were categorized based on whether they corresponded to the side of symptom onset (severe) or the opposite side (moderate) and compared for differences in gene expression. We employed two *a priori* approaches; first we identified genes differentially expressed between PD and controls and between PD brain hemispheres. Second, we examined the presence of, and correlates to, variations in the asymmetry for some differentially expressed genes. We found large variation among individuals with PD, and so PD stratification by gene expression signature was required for patterns of genetic asymmetry to emerge. For a subset of PD brains, hemispherical variation of *CCT* gene levels correlated with the side of PD symptom onset. In a mouse model of PD, neurons with *α*-synuclein inclusions had decreased *Cct* expression. These results suggest that *CCT* expression plays a protective role in PD.

## Introduction

Asymmetric presentation is a well-documented aspect of many neurodegenerative diseases (Cubo et al., 2010; Lubben et al., 2021). Parkinson’s disease (PD) motor symptoms typically present asymmetrically, and the initially impacted side retains a more severe presentation as disease progresses (Cubo et al., 2010; Steinbach et al., 2023). A recent review of 80 studies explored how lateralized motor symptoms affect non-motor outcomes in PD (Voruz and Peron, 2025). This asymmetry can also be seen in imaging of dopamine transporters or in diffusion MRI conducted as part of diagnosis (Filippi et al., 2005; Xiao et al., 2021). These measures of dopaminergic terminal loss and atrophy are more severe in the brain hemisphere contralateral to motor onset (Djaldetti et al., 2006).

Notably, the hemisphere affected first in PD is not entirely random. There is a small but significant bias for onset side to correspond to the dominant hand (Barrett et al., 2011; van der Hoorn et al., 2012). Any degree of non-randomness implies some intrinsic or extrinsic brain hemisphere differences influence the onset and/or progression of PD. In other words, persistent nonrandom asymmetry suggests that one hemisphere is more protected before disease onset, or becomes relatively protected (i.e., is less burdened) after the disease manifests. Identifying the mechanisms underlying either or both scenarios would be valuable for diagnostic and treatment strategies.

There are two possibilities for nonrandom PD asymmetry, which are not mutually exclusive (Cubo et al., 2010). First, intrinsic differences or systematic lateralized environmental exposures may make one hemisphere more vulnerable to disease. Second, disease itself may induce differences between hemispheres. Vulnerability to PD may be equivalent between hemispheres at some arbitrary developmental stage, but in the absence of a synchronizing mechanism, asymmetry could persist or become more exaggerated following onset. Stochastic processes likely lead to subtly different disease burden to either hemisphere. However, initially random effects could accumulate and potentially lead to amplifying feedback due to the dynamic interaction between localized and global disease responses. For example, a protective stress response or neurotrophic signal might be stronger in the less affected hemisphere. Alternatively, one process with both positive and deleterious effects, such as inflammation, could have differential effects between hemispheres.

We are not aware of any study comparing the gene expression landscape of intra-hemispheric differences in PD. To fill this gap, we obtained matched left and right frontal cortical samples from PD cases and age- and sex-matched neurologically-normal cases. We also had relevant clinical information including the side of PD symptom onset in the diseased cases. In the present study we sorted neuronal nuclei from these tissues and performed bulk RNA-seq to identify differences in gene expression related to disease or brain hemisphere. The questions we sought to address were:

- Is there differential gene expression between brain hemispheres in PD that relate to the side of symptom onset?
- How does each PD hemisphere compare to control brains?
- Are there any gene expression differences involved in brain lateralization or other early processes, that are likely to predispose one side to PD symptom onset?

## Materials and methods

### Study cohort

Frozen tissue samples were obtained through the NIH NeuroBioBank from the Human Brain and Spinal Fluid Resource Center in Los Angeles, CA and from the University of Miami Miller School of Medicine with approval from the IRB of the Van Andel Research Institute (IRB #15025 as non-human subjects’ research). Each anonymized case included information on the donor’s age, sex, ethnicity, and cause of death. Additionally, the tissue post-mortem interval, duration of disease, side of onset, and neurofibrillary tangle burden (Braak stage) (Braak et al., 2003). Neurons of the prefrontal cortex were selected for this study, as this region shows pathology only at very advanced stages of PD and would be unlikely to show indicators of local neuronal loss. We deliberately choose PFC since the early Braak stages of the disease leave it relatively intact. Therefore, expression changes between hemispheres are likely unrelated to neurodegeneration and more likely due to gene expression ontology. Matched hemisphere samples from 5 female and 5 male healthy control, and 9 female and 28 male PD brain samples were available for analysis. Braak stages ranged from 1.5 to 6 with a mean of 2.5. The average age at death was 76.1 years. Sample specific details are included in Supplementary Table 1.

### RNA-sequencing

Neuronal nuclei were isolated from frozen and unfixed human prefrontal cortex by lysing the tissue then tagging with an anti-NeuN antibody and using flow cytometry (Matevossian and Akbarian, 2008; Li et al., 2019). Nuclear mRNA has been previously demonstrated to provide a good representation of total cellular mRNA (Lake et al., 2017). cDNA libraries were prepared by the Van Andel Genomics Core from 500 ng of total RNA using the KAPA RNA HyperPrep Kit using RiboseErase. RNA was sheared to an average of ~300 bp. Individually indexed cDNA libraries were pooled, and 75-bp paired-end sequencing was performed on an Illumina NextSeq 500 sequencer. Base calling was done by Illumina NextSeq Control Software (NCS; v2.0), and the output of NCS was demultiplexed and converted to FastQ format with Illumina Bcl2fastq (v1.9.0).

### Differential expression analysis

Following sequencing, fastq files were trimmed using Trimmomatic V0.36 and the settings: ILLUMINACLIP: TruSeq3-PE.fa:2:30:10 LEADING:3 TRAILING:3 SLIDINGWINDOW:4:15 MINLEN:36. Trimmed fastq files were aligned to HG19 using STAR v2.7 (Dobin and Gingeras, 2016). Alignments (bam files) were converted to feature counts using HTSeq v0.6.0 referenced against the ENSEMBLE annotation of HG19: Homo_sapiens. GRCh37.87.gtf counting against the feature “exon,” grouped by “gene_id,” and using the strand parameter “reverse.” Technical replicates were combined for samples with duplicates. Gene counts were normalized using edgeR (TMM) and tested for significant differential expression with Limma and Voom, in R (v4.10.2) (Robinson and Oshlack, 2010). Initial differential expression was estimated with a design of Gene expression = ~0 + combined category (PD or hcntrl, sex, side of onset, hemisphere) + duration of illness + library RIN + library size + difference relative to mean age + positive nuclei per mg tissue + PMI. This comparison was used to identify the least statistically different 2,000 genes for healthy control vs. PD. The function RUVr via Ruvseq (Risso et al., 2014) was used to estimate 5 correcting variables to minimize residuals based on model: Gene expression = ~0 + combined category + duration of illness + difference from mean age. These 5 normalizing variables were added to the second model for final analysis. For comparison of display of expression values the normalized count from RUVseq was used, though statistical analysis was based solely on log CPM from Limma.

Data are deposited at NCBI GEO: GSE264050.

### Enrichment analysis

Enrichment of ontology for gene sets was done using ranked enrichment by FDR with GSEA (Subramanian et al., 2005), or for gene set enrichment by STRING V.12 (Szklarczyk et al., 2023).

### Publicly accessed data description of the mouse experiment

The methods for the original study are extensively described in the source manuscript (Goralski et al., 2024). A brief description of the methods which produced the data used in this study are provided here. At 3 months of age, C57BL/6J mice were injected into the dorsal striatum with recombinant mouse *α*-synuclein pre-formed fibrils (PFFs) via stereotactic surgery. Mice were aged 3 months post-injection prior to humane euthanasia, perfusion, and brain harvesting. Harvested brains were fixed overnight in 4% PFA. After fixation, brain tissue was processed into paraffin and sectioned at 6 μm. Finally, whole transcriptome spatial transcriptomics was performed on sections using the GeoMx^®^ Digital Spatial Profiler. Differential expression analysis was performed using a mixed effects linear regression model. The code to reproduce the presented analysis is publicly available here: https://github.com/Goralsth/Gene_expression_asymmetry_in_Parkinsons_Disease_Variation_of_CCT.

## Results

We sought to identify potential gene expression differences between the more affected (severe) and less affected (moderate) hemispheres in PD brain tissue and in comparison, to healthy control samples. We performed bulk-RNA-sequencing on neuronal nuclei (NeuN+) derived from frontal cortex tissue of 28 PD brains and 10 healthy control brains. Matched samples from the left and right hemispheres of each brain were used. Matched hemisphere RNA-seq for 5 female and 5 male healthy control (average age 78, 69 years, respectively) were compared to data from 9 female (3 left onset, 5 right onset, 1 bilateral; average age, 77 years) and 28 male (9 left onset, 8 right onset, 2 bilateral; ave. age, 77 years) samples. The average Braak stage for PD brains ranged from stage 2–3.

**Figure 1:**
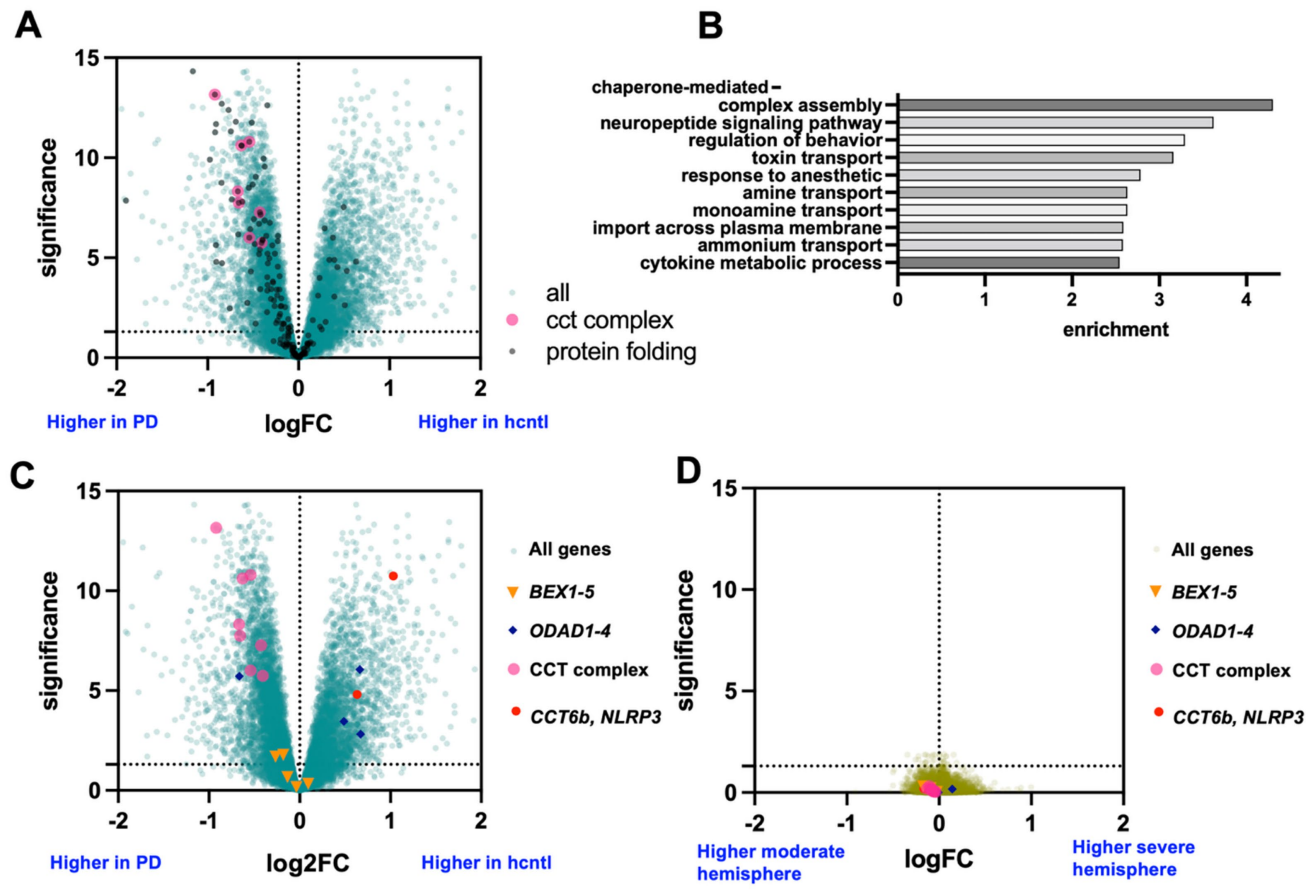
Differential gene expression across entire sample set. **(A)** Volcano plot of DE comparing all PD samples vs. all healthy control samples (hcntl). All genes annotated with the biological process GO category “protein folding” are indicated in black, the eight primary subunits of the CCT complex in magenta. **(B)** The most highly enriched GO categories of all significant (FDR > 0.05) genes. **(C)** Volcano as in **(A)**, but with four groups of genes indicated for use as marker posts and genes of interest to compare across different analysis. **(D)** Volcano plot of DE comparing asymmetry (severe vs. moderate hemisphere) across all PD samples.

We compared the gene expression levels in PD patients to that of healthy controls according to sex, both jointly and separately and including covariates for age, duration of illness, and 5 correcting variables. We found large expression changes (Figure 1A). As expected, both sex specific and non-specific PD changes were identified. The most prominent disease-related processes, based on gene set enrichment, were driven by the expression of CCT chaperonin complex subunits (Figures 1A,B; Supplementary Figure S1A). The *CCT* genes encode for proteins that form the CCT chaperonin complex, which has been reported to be associated with PD risk as well as being critical in PD processes including *α*-synuclein aggregation (Zhen et al., 2017; Grantham, 2020; Noori et al., 2023). In addition to its ability to form toxic aggregates (Surguchov and Surguchev, 2022), variations in α-syn expression between the lesioned and unlesioned hemispheres have been reported in a mouse model of Parkinson’s disease (Weetman et al., 2013). These CCT genes, as well as other chaperone protein and protein folding related genes, were generally elevated in PD patients compared to healthy controls. Genes down-regulated in PD brains were less enriched for specific biological processes, but included axoneme and dynein linked genes, including *ODAD1-4* (Figure 1; Supplementary Figure S1B).

The central question of this study was what (if any) gene expression is statistically different across hemispheres relative to PD side of onset. In the first instance, we found little evidence that any genes were significantly asymmetrical by comparing severe to moderate hemispheres across all patients and by linear modeling including age, duration of illness, and five composite variables created to remove unwanted variation (methods) (Figure 1D). A few genes were significant for males or females when analyzed separately (9 and 40 respectively, Supplementary Figure S1C).

**Figure 2:**
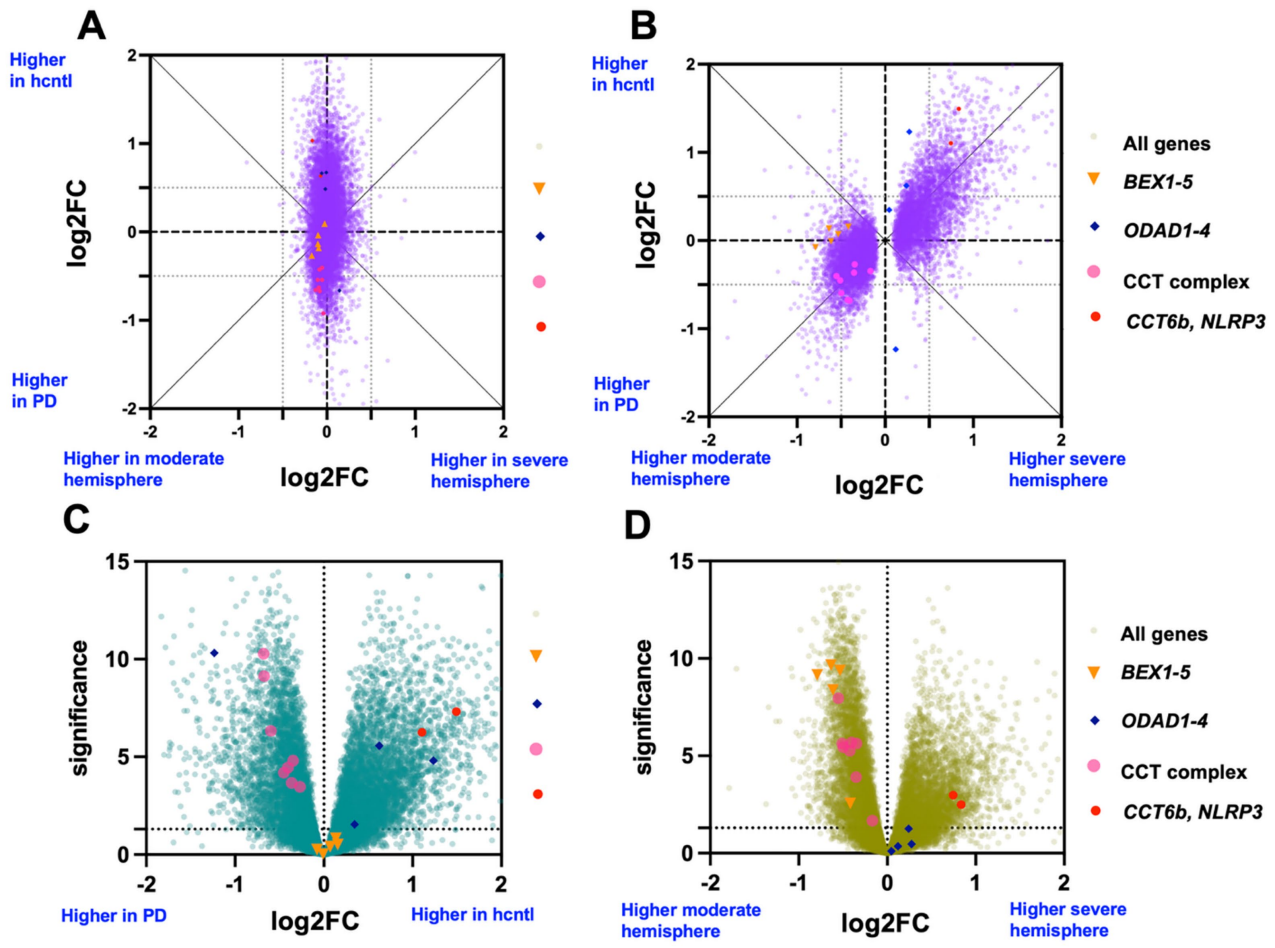
Differential gene expression across a subset of the most highly correlated PD brains (by hierarchical clustering). In purple are comparison of DE changes by disease state vs. asymmetry. GLM estimated log2 fold change of two separate comparisons: healthy control vs. PD (Y-axis) against severe hemisphere vs. moderate hemisphere of PD brains (X-axis). **(A)** Entire set of 25 PD brains, **(B)** subset of 14 least variable and clustered PD brains. Genes with FDR < 0.05 in asymmetry are plotted. **(C)** As in Figure 1C except the comparison PD brain samples are only the 14 least variable PD brains. **(D)** As in Figure 1D except the comparison hemispheres derive from only the 14 least variable PD brains.

Given this generally negative asymmetrical result, we sought further clarification to support or reject the initial observation that no asymmetrically expressed genes were present. The lack of straight-forwardly significant asymmetry across the 25 (unilateral onset) PD brains could reflect the large variation between people compared to near identity across hemispheres within a given individual. Of course, a large enough study size may help alleviate this issue. As a more tractable tactic to further address variation between subjects, we asked whether any asymmetry was apparent after selecting only the most similar PD patients (based on gene expression levels) for comparison with controls. We clustered samples using rank correlation of the top 2,500 most variable and highly expression genes. On this basis selected paired samples from the most similar cluster of PD-only brains, consisting of 14 PD brains with both hemispheres (8 male, 6 female) were compared to the 10 control brain samples (Supplementary Figures S2, S3). In this narrower comparison, differentially expressed (DE) genes for PD vs. healthy control remained like the full comparison. However, unlike in the full PD group among a subset of similar PD brains, we identified many significant DE asymmetry genes comparing severe vs. moderate hemispheres (Figures 2C,D).

Of the 9,274 significant DE asymmetry genes in the clustered subset, 61% were also DE disease specific genes, and of those 93% showed the opposite direction of fold-change, 7% the same direction, i.e., most genes asymmetric and higher in the severe hemisphere relative, were downregulated in PD relative to control. This is unexpected, as we predicted that the expression signature in the *hemisphere* with greater PD symptoms would correspond to the expression of the *population* with greater PD symptoms. At least in this set of most similar PD brains that relationship is reversed. Why the expression changes are inverted relative to expectation demands further discussion. However, disregarding, for now, directionality, the high correlation suggests that the same processes are affected in both comparisons. That is: genes that distinguish a severely affected hemisphere from a moderately affected hemisphere in a PD patient are largely the same as those which distinguish a healthy vs. a PD brains more generally.

Conversely, we searched for genes that were clearly affected by (or predicted by) the side of onset but not disease status (DE asymmetry genes that were significantly asymmetric and showed at least a 0.5 log_2_ fold change (FC) *difference* in effect size between comparisons). These genes were not plentiful but were enriched for brains that expressed x-linked (*BEX*) family genes. For the *BEX* genes identified, no difference in expression was seen for PD vs. control, but these were more highly expressed in the moderate hemisphere compared to the severe hemisphere. It is therefore possible that *BEX* gene expression is associated with which hemisphere first develops PD symptom onset. The resulting hypothesis is that *BEX* gene expression may be partially protective in delaying PD progression to one hemisphere, while having no impact on whether a person develops PD or not. These expression levels were higher in the less severe hemisphere, though due to the small number of PD brains examined, we cannot distinguish PD side onset DE from left vs. right brain hemisphere differences alone. These *BEX1-5* were only identified within a small subset of PD patient brains selected due to overall RNA-seq similarity.

Some genes were more equally expressed between hemispheres than expected based on disease DE. That is, some genes that differed between healthy and disease brains appeared similar across hemispheres. Most enriched in this category were dynein genes, including 3 of 4 *ODAD* genes. Dynein related genes were DE according to disease status in both the entire comparison as well as the filtered subset.

**Figure 3:**
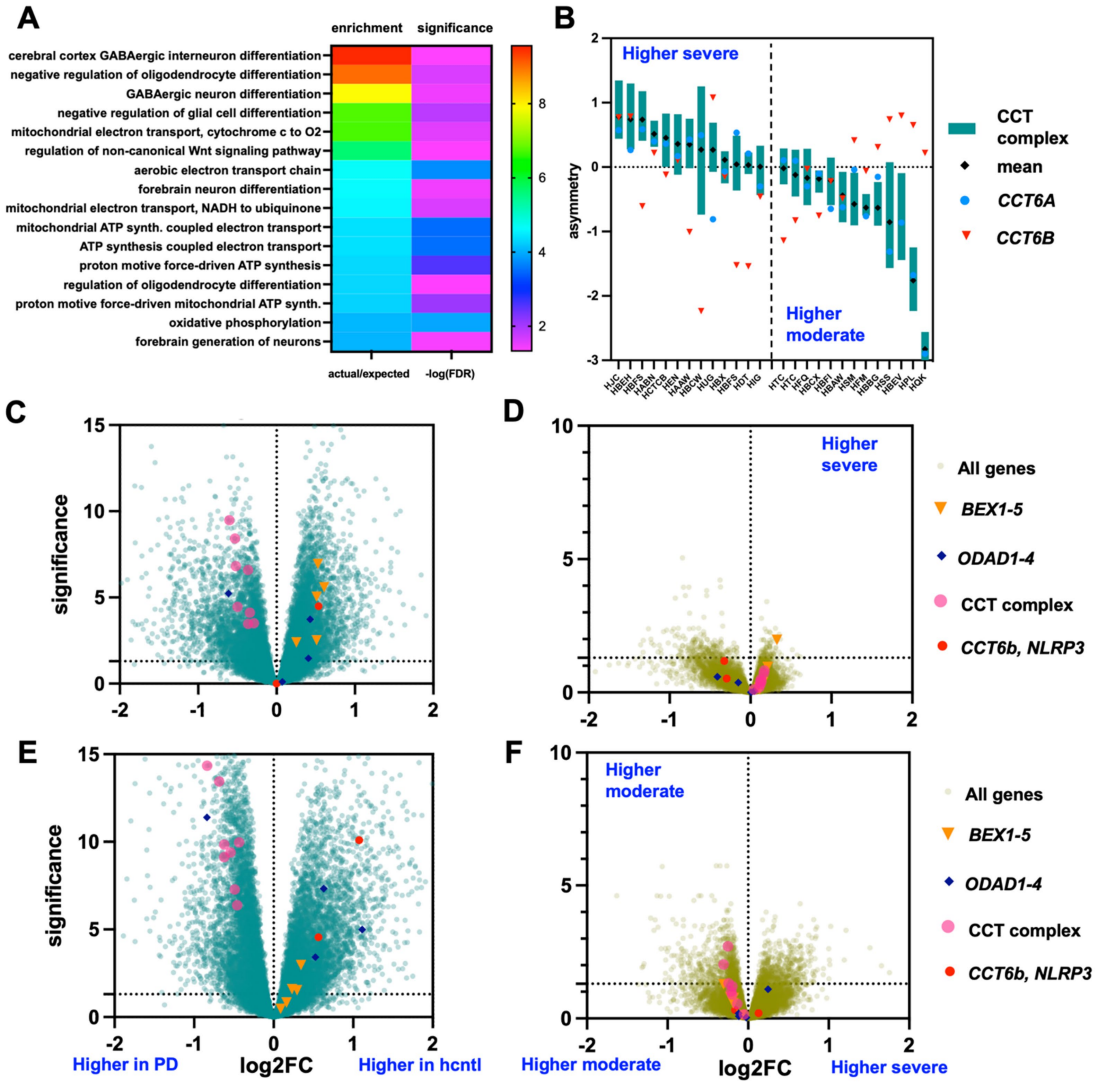
Categorizing PD brains by directional asymmetry of CCT gene expression. **(A)** Gene set enrichment of genes with a significant association by GLM with the average expression of the 8 CCT subunit genes. **(B)** Relative expression between hemispheres of all 25 PD brains (2 samples are replicated in the plot, bars represent mean and standard deviation of the 8 CCT genes by zscore exp. severe—moderate). To the left of the vertical dashed line are the samples with higher CCT expression in the severe hemisphere, to the left those with higher in the moderate hemisphere. **(C)** Volcano of (CCT higher in severe)-PD vs. all controls. **(D)** Volcano of severe vs. moderate expression levels in the (CCT higher in severe)-PD set. **(E)** Volcano of (CCT higher in moderate)-PD vs. all controls. **(F)** Volcano of severe vs. moderate expression levels in the (CCT higher in moderate)-PD set.

As well as classifying asymmetry genes according to known PD disease associations, we also proceeded in the other direction: we looked for patterns of asymmetry in PD gene expression levels. We considered whether variation in asymmetry of PD gene expression could correspond to differences in disease states. The most statistically enriched PD related biological processes in this study included the genes encoding the chaperonin CCT (also known as the TriC complex). These genes were more highly expressed in PD patients vs. controls, especially in males. By generalized linear modeling we found that genes correlated with *CCT* expression were enriched for several types of neuronal differentiation and metabolic pathways (Figure 3A). This suggests that different levels of CCT could correspond to processes important in PD resistance or protection. Indeed, in examining the distribution of *CCT* expression between hemispheres in the complete set of samples, there appeared to be a trend toward elevated expression of *CCT* genes in the less effected PD hemisphere (Supplementary Figures S1F,G). This relationship was not specific to *CCT* though, and overall, there was a nonsignificant trend: the more strongly disease-associated a gene expression level was on average, the more likely it was to be expressed more in the moderately affected hemisphere. Still, these observations suggest that a pattern of asymmetry may be present in the PD genes identified.

We noticed one other interesting feature in *CCT* expression related to the alternative subunit, *CCT6b*. The significant disease associated *CCT* genes we identified include *CCT6b* which has previously been reported to be inversely correlated with the other *CCT* gene subunits, especially with respect to cancers (Li et al., 2021; Shemesh et al., 2021). In agreement with this observation, in the present study, genes *CCT1-8* were more highly expressed in healthy control tissue whereas *CCT6b* was proportionally less expressed relative to PD patient brains. But comparing across hemispheres within patients, *CCT6b* was on average similarly expressed to the rest of the *CCT* subunits, apparently pointing to an inverse correlation by disease but not hemisphere (Figure 3B). When measured explicitly, there was, in fact, a negative correlation by cpm (*r*^2^ = −0.44) or no correlation according to normalized counts (*r*^2^ = 0.0) of *CCT6b* with the primary subunits compared to high positive correlation between the primary *CCT* genes (cpm: *r*^2^ = 0.93, normalized: *r*^2^ = 0.85). The explanation is, of course, that whereas the average expression of the *CCT6b* and the primary *CCT* subunits were similar, for given PD individuals the expression profiles were often negatively correlated, but with many people showing asymmetry in one direction and other in the opposite direction (Figure 3B). That is, there was a continuum to the expression of *CCT* genes, with some PD patients showing increased expression in the severe hemisphere and some showing increased *CCT* expression in the moderate hemisphere, with the alternative subunit *CCT6b* being weakly to negatively correlated correspondingly. We asked first whether statistically significant gene expression asymmetry was present after stratifying PD patients according to *CCT* gene expression asymmetry. Incidentally this method of categorization produced a different grouping than the clustering-based approach in Figure 2.

After stratifying all PD patients based on the directionality of *CCT* gene asymmetry, similar DE was observed for both types compared to healthy controls. However, significant asymmetry in gene expression was seen for both groups, with many genes having opposite directions of asymmetry and other genes showing asymmetry in one group but not the other (Figures 3C–F). Comparing the gene set enrichment of significant genes produced different ontologies (Supplementary Figure S4).

**Figure 4:**
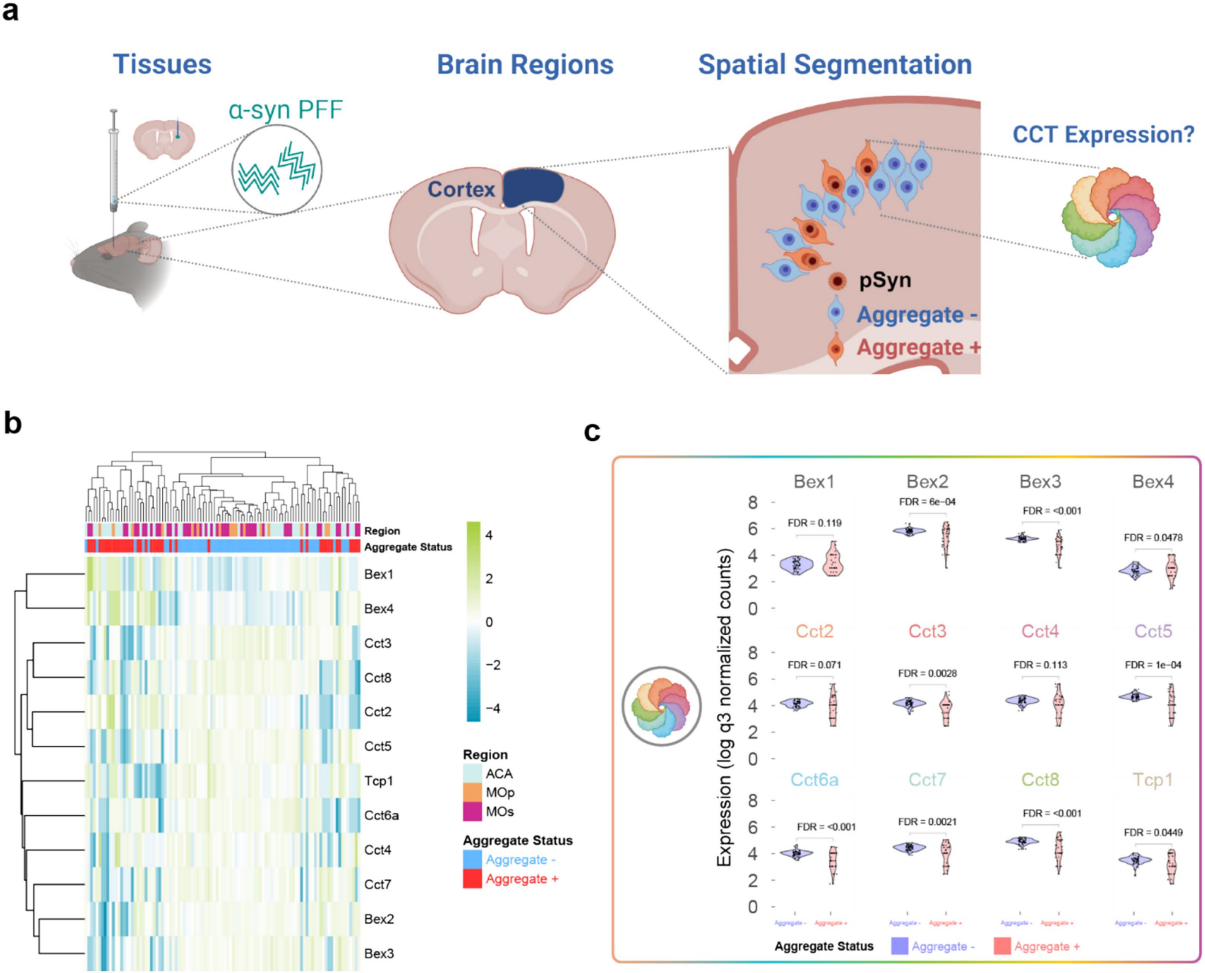
Exploring TRiC expression in Lewy-like aggregate and non-aggregate bearing neurons. **(A)** Cartoon graphic depicting the experimental paradigm for exploring CCT expression in aggregate and non-aggregate beating neurons. **(B)** Heatmap of genes identified as differentially expressed in human asymmetry data from the PFF-injected mouse data. **(C)** Violin plots showing expression levels of human asymmetry DE genes between aggregate and non-aggregate bearing neurons from the mouse PFF injection data.

Given the overall variability in the human data, and the inability to perform experimental manipulations with the human material, we next sought to cross-validate the asymmetry findings using a publicly available data source from mice. The original public dataset was generated in a study that aimed to identify molecular changes induced by misfolded *α*-synuclein pathology in the cortex of mice tissue injected with α-synuclein pre-formed fibrils (PFFs) (Goralski et al., 2024). Data was sourced from the following public repository: https://zenodo.org/records/10729767. Of the identified human asymmetry genes *Bex1, Bex2, Bex3, Bex4, Tcp1, Cct2, Cct3, Cct4, Cct5, Cct7*, and *Cct8* were found in both datasets. We visualized expression of these genes using a heatmap and hierarchical clustering (Figure 4B). The hierarchical clustering reveals that aggregate bearing cells can be differentiated from non-aggregate bearing neurons by the expression of asymmetry genes alone, suggesting strong differences in expression. Interestingly, all *CCT* genes typically show higher expression in non-aggregate bearing cells. Aggregate bearing neurons generally show downregulated expression of *CCT* genes compared to non-aggregate bearing neurons. The significance and effect size of these pattens is further visualized using violin plots (Figure 4C). The differential expression values were publicly available and were originally computed using a linear mixed effects regression model. Of the *CCT* genes *Tcp1, Cct3, Cct5, Cct6a, Cct7, and Cct8* were significantly downregulated (FDR < 0.05) while *Cct2*, and *Cct4* were not significant (FDR > 0.05). *Bex2, Bex3*, and *Bex4* were significantly differentially expressed (FDR < 0.05) whereas *Bex1* was not significant.

## Discussion

In classic twin studies, genetic, epigenetic, and environmental differences are controlled; this allows for the precise examination of the remaining non-concordant phenotypes (Sahu and Prasuna, 2016). These studies are also the gold standard for estimating the genetic heritability of traits and the relative contribution of environmental vs. innate risk factors (for PD around 30%). Incidentally, while these studies show higher concordance in monozygotic twins vs. dizygotic ones (one example 20 vs. 13, Goldman et al., 2019; review: Hagenbeek et al., 2023), no measurement of side of onset concordance has yet been done. Each hemisphere of a brain is a near identical copy of the other, differing only subtly (genetically and anatomically) due to functional lateralization and minor differences in environmental exposures. General brain hemispheric asymmetry in neurodegenerative diseases was recently reviewed in a publication by our group (Lubben et al., 2021). It formed the basis for the present examination of differences in gene expression between hemispheres within PD patients and in comparison, to healthy control brain samples. In the present study we find that side of onset is partially correlated with differences in gene expression even in regions not yet directly affected by the disease.

Three classes of genes were identified. These include: DE genes associated with both Parkinson’s disease and hemisphere relative to side of PD onset (including the *CCT* genes), DE genes associated with disease status but not hemispherical levels (including dynein genes and *ODAD1-4*), and DE genes associated with hemisphere but not disease (including *BEX1-5*). These classes support the possibility that some biological processes are distinctly asymmetrical before and/or during the etiology of the PD.

We found that expression of the *CCT* genes which encode the TRiC complex are associated with PD. The TRiC complex is required for proper folding of tubulin, is a modulator of protein aggregation, and may impact immune processes (Martin-Cofreces et al., 2021). Brain expressed x-linked (*BEX*) genes are highly expressed in neuronal tissue, have been linked to stem cell capacity, and are upregulated in brain tumors cancers (Alvarez et al., 2005; Ito et al., 2014; Wang et al., 2021; Aisa et al., 2022). Both gene sets were more highly expressed in the less affected hemisphere of similar PD brains. The variation could be either protective or indicative of reduced damage by the disease.

Counter to expectations, the *CCT* genes showed higher expression in the less affected hemisphere rather than in the severe hemisphere, even though overall CCT expression was lower in healthy controls than in PD cases. This paradox could be resolved in various ways. For instance, CCT-mediated processes could be both compensatory at a local level and unregulated globally in response to PD. Thus, one hemisphere could exhibit slower progression due to greater levels of *CCT*. Interestingly, control female brains had high *CCT* expression levels while healthy male brains had low levels. In PD brains, *CCT* expression was roughly the same for male and females and more like control female levels. This suggests that CCT might be protective at a certain level with healthy females already expressing that level, but males have “room” for increased expression. Men have from 1.5 to 2-fold the rate of PD diagnosis as women (Wooten et al., 2004), further supporting a protective role for higher *CCT* levels. Our results are of particular interest in the context of recent reported results that individuals with pathogenic variants in the CCT complex develop brain malformations, intellectual disability and seizures (Kraft et al., 2024). Due to the conserved mutation of *CCT* genes, these disorders were termed “TRICopathies.” Here, we extend the protective effect to higher levels of wildtype CCT in PD.

We conjecture that at least 2 PD subtypes are present in the patients in this study, and these can be typed according to *CCT* expression patterns. One subtype has increased expression in the severe PD hemisphere the other increased expression in the less affected hemisphere. Both correspond to a set of differentially expressed but different genes. A speculative biological explanation does require a somewhat complicated model where the CCT chaperonin is involved in both PD types but in different ways or to different degrees. This is possible and hypothetical scenarios can be readily imagined. For instance, one PD type may be driven by *α*-synuclein misfolding, which is impeded by compensatory chaperonin presence, whereas in another increased neural cell body growth or even limited proliferation is associated with lower levels of CCT overall and increased levels of *CCT6b* expression.

Among the healthy control brains, the inverse relationship for *CCT6b* expression and the primary CCT subunits was also present, though to a lesser degree, 4/10 vs. 12/24 for the PD case brains. However, the degree of asymmetry for these genes was greater in the PD brains. It may be that asymmetry is present but is exaggerated following disease onset; alternatively, greater asymmetry may make PD more likely. This remains highly speculative at this stage.

Gene expression differences between the severe and moderate hemispheres within PD patients were identifiable following stratification of PD patients. Most of these genes correspond to and were also identified by comparing PD brains vs. healthy controls in general. We suspect the origin of this pattern could be related to compensatory effects, which are initiated in a disease state, but which are more apparent in the less effected hemisphere due to less large-scale disruption. PD brain samples were from prefrontal cortex and from relatively early-stage PD, mostly Braak stages 1–3 (a single stage 5, and 1 stage 6) (Braak et al., 2003). We would not expect severe neurodegeneration in this region for these patients. Thus, we hypothesize that important cellular stress related processes can both permeate the entire brain and/or be partially bound to the originating hemisphere.

PD is a complex neurodegenerative disorder with only partially understood underpinnings. Ultimately the present study compared gene expression from 28 patients and 10 control brains. Meaningful comparison between hemispheres within patients required additional stratification of PD cases. Limited biographical information associated with the samples unfortunately precludes analysis or even speculation regarding what else, beyond gene expression patterns, differentiated these groups of patients. We also cannot extrapolate how representative these patients may be for PD patients overall. For these patients though, layering a comparison across hemisphere onto the greater analysis of disease required additional grouping. This could be taken to imply that PD is composed of distinct subtypes or even that the disease has intrinsically unique features for every individual. Here *CCT* genes seemed useful for discrimination. More generally, asymmetry related measurements may become useful for PD research and clinical diagnosis, but only in the context of careful filtering and calibration of disease subtypes and individual variation.

To better understand the role of CCT expression in PD, we sought to validate our findings using an independent, publicly available dataset from a mouse model of misfolded *α*-synuclein pathology, a hallmark of PD. In the original study that generated this dataset, the molecular dysfunction caused by misfolded α-synuclein was comprehensively characterized in both a mouse PFF injection model and human postmortem tissue, revealing an overlapping molecular signature in both systems (Goralski et al., 2024).

Using this dataset and the accompanying analysis, we found that several components of the CCT complex, including *Tcp1, Cct3, Cct5, Cct6a, Cct7*, and *Cct8*, exhibited significantly reduced expression in neurons with α-synuclein inclusions. However, it remains unclear whether the loss of CCT expression is a downstream consequence of the toxicity of aggregates or whether lower expression of CCT genes increases vulnerability to α-synuclein pathology.

The alignment of our initial findings with those observed in this rigorously controlled mouse model suggests a potential role for the CCT complex in either mediating cellular vulnerability to, or responding to, the molecular mechanisms of neurodegeneration in PD. Future research should aim to clarify whether the CCT complex contributes to the predisposition to misfolded α-synuclein or plays a role in mitigating its effects.

## Conclusion

For a subset of PD brains, hemispheric increased *TRiC/CCT* gene levels *protectively* correlate with the mild side of PD symptom onset, likely due to its chaperonin functions in protein folding, especially tubulin, vital in neurite projections. This result is in line with recent data that indicate loss-of-function mutations in any of the 8 CCT subunits of the TRiC-chaperonin complex lead to individuals with brain malformations, intellectual disability, and seizures. So, the question is whether such gene expression levels are the cause or consequence of PD and other brain abnormalities and/or brain lateralization; we suspect the former. Stimulating CCT levels could be beneficial for some PD patients, but given the low levels in healthy controls, the relationship may be more complicated. We cannot differentiate between possibilities wherein CCT expression variation is a pre-existing risk factor or whether CCT expression changes in response to early disease signals. Untangling this issue will inform diagnostic and treatment strategies.

## Data Availability

The datasets presented in this study can be found in online repositories. The names of the repository/repositories and accession number(s) can be found at: GSE264050.
