# Supplementary material for: Gene expression asymmetry in Parkinson’s disease: variation of CCT gene expression is correlated with hemisphere specific severity
Source: Front Mol Neurosci. 2026 Jan 13;18:1743557. doi: 10.3389/fnmol.2025.1743557 (PMC12835222; doi:10.3389/fnmol.2025.1743557)
Supplement: Supplementary file 1 [file Data_Sheet_1.docx]

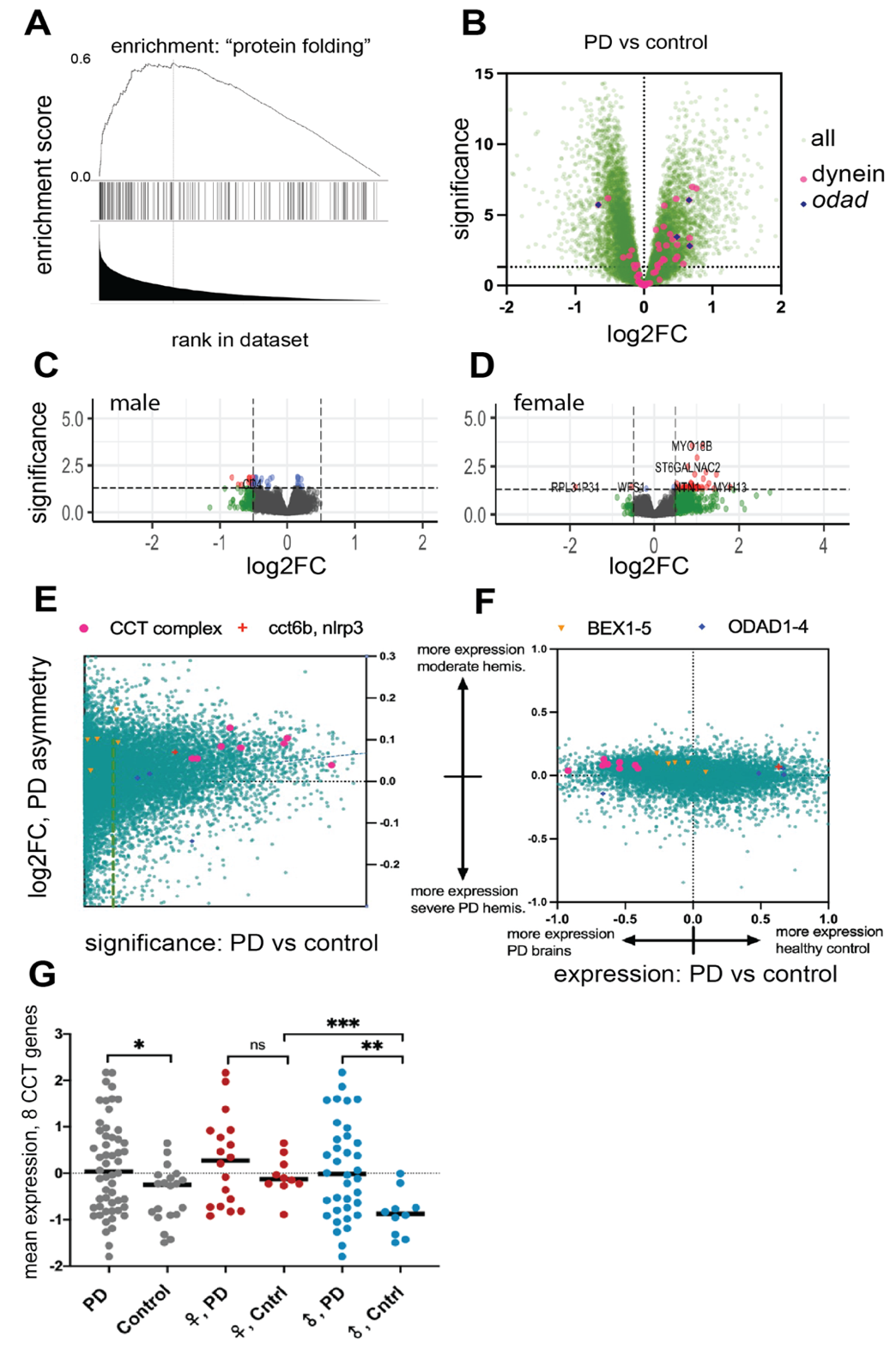


***Supplemental figure 1. A) ranked gene set enrichment analysis, ordering is by significance comparing PD to healthy controls. B) Volcano plot of PD vs healthy controls with all genes named or related to dynein indicated in magenta. C) Volcano plot of male PD brains: severe vs moderate hemisphere. D) Volcano plot of female PD brains: severe vs moderate hemisphere. E) asymmetry log2FC (y-axis) vs significance (-log10 adj. p.value) of PD vs healthy control. F) asymmetry log2FC (y-axis) vs. log2FC difference healthy vs. PD. G) comparison of CCT gene expression. Expression is in Z-score normalized RUV-seq adjusted counts.***


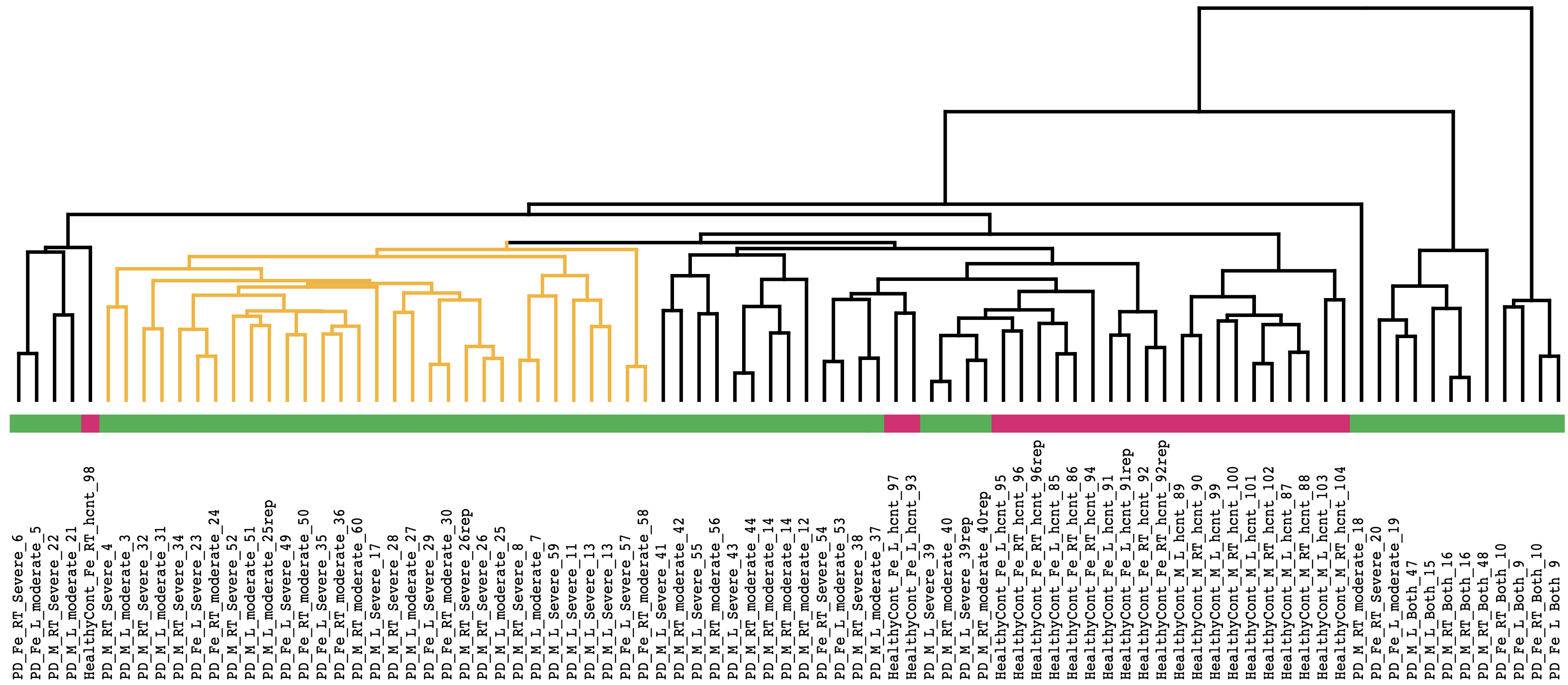


***Supplemental figure 2. Clustering (by average) of all samples by rank correlation of the top 2,500 most variable high expression genes. The most similar 14 PD patients (8 males, 6 females, indicated in yellow) were compared to 10 control brain samples.***


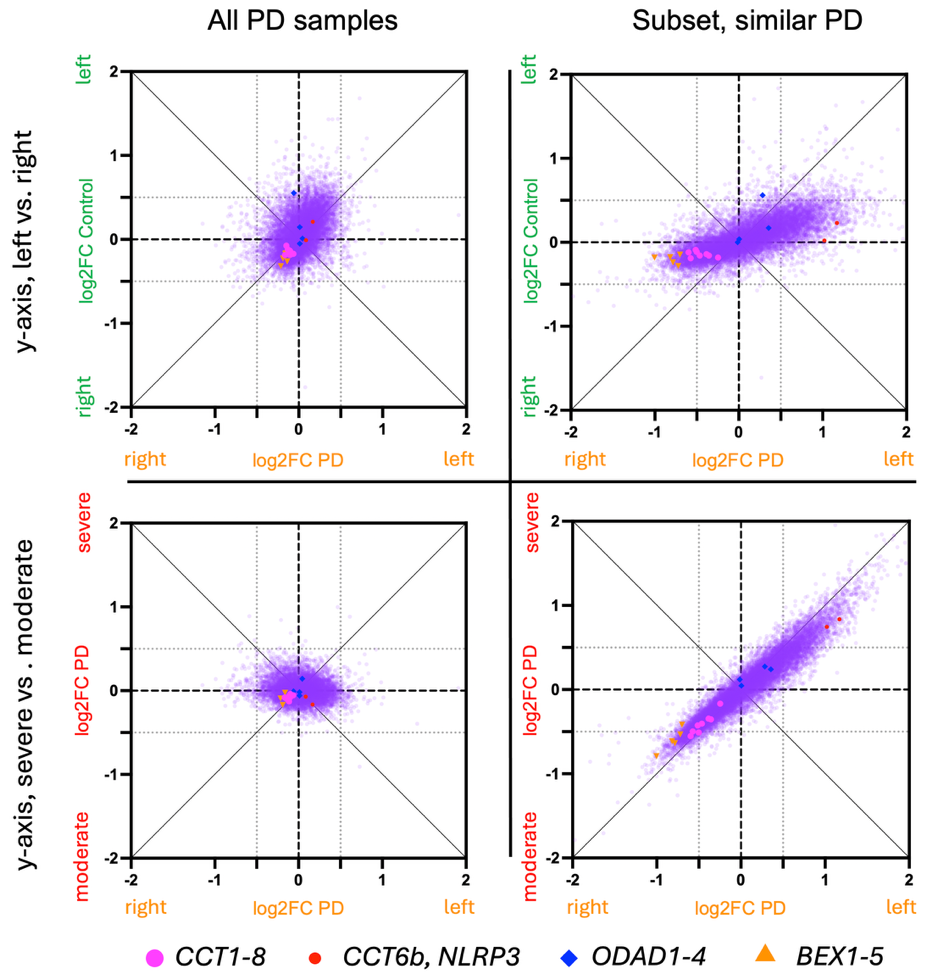


***Supplemental figure 3. Log2 fold-change plots, the left column consists of results for all PD samples, and the right column represents only the subset of clustered and similar PD samples. The top row shows healthy control sample gene expression fold change comparing left vs. right hemispheres for each gene relative to PD sample gene expression fold change for left vs. right hemispheres. The bottom row shows PD severe vs. moderate hemisphere gene expression fold change relative to PD left vs. right hemisphere gene expression fold change.***


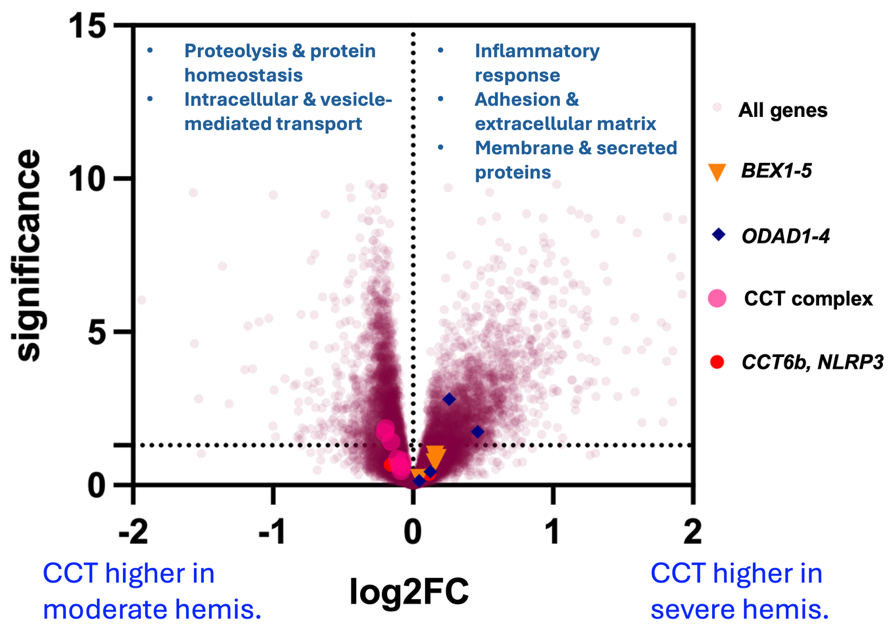


***Supplemental figure 4. Volcano plot comparing the gene expression differences between two subsets of PD patients: those with asymmetrically higher* CCT *expression in the severe PD hemisphere vs. those with higher* CCT *expression in the moderate hemisphere. The functionally enriched terms in each direction based are summarized, with the expression signature of PD patients with higher CCT expression in the moderate hemisphere showing upregulation of proteostasis and the PD patients with higher expression in the severe hemisphere showing a greater immune and inflammatory effects.***
